# Dr. Charles Francis Clowe

**Published:** 1912-06

**Authors:** 


### OBITUARY

Dr. Charles Francis Clowe of Schenectady, died April 29, 1912, aged 46. He was a graduate of Albany, 1888.

